# Autosomal dominant polycystic kidney disease and pioglitazone for its therapy: a comprehensive review with an emphasis on the molecular pathogenesis and pharmacological aspects

**DOI:** 10.1186/s10020-020-00246-3

**Published:** 2020-12-11

**Authors:** Aryendu Kumar Saini, Rakesh Saini, Shubham Singh

**Affiliations:** 1 Department of Pharmacy, Chaudhary Sughar Singh College of Pharmacy, Etawah, Uttar Pradesh India; 2 Department of Pharmacy, Shri Ram Lakhan Tiwari College of Pharmacy, Etawah, Uttar Pradesh India

**Keywords:** PPAR gamma, Cystic fibrosis, Polycystin-1, Hedgehog pathway, JAK2 protein, MAP kinase signaling system, Platelet endothelial cell adhesion molecule-1

## Abstract

Autosomal dominant polycystic kidney disease (ADPKD) is an inherited chronic kidney disorder (CKD) that is characterized by the development of numerous fluid-filled cysts in kidneys. It is caused either due to the mutations in the PKD1 or PKD2 gene that encodes polycystin-1 and polycystin-2, respectively. This condition progresses into end-stage renal disorder if the renal or extra-renal clinical manifestations remain untreated. Several clinical trials with a variety of drugs have failed, and the only Food and Drugs Administration (FDA) approved drug to treat ADPKD to date is tolvaptan that works by antagonizing the vasopressin-2 receptor (V2R). The pathology of ADPKD is complex and involves the malfunction of different signaling pathways like cAMP, Hedgehog, and MAPK/ERK pathway owing to the mutated product that is polycystin-1 or 2. A measured yet substantial number of preclinical studies have found pioglitazone to decrease the cystic burden and improve the renal function in ADPKD. The peroxisome proliferator-activated receptor-gamma is found on the epithelial cells of renal collecting tubule and when it gets agonized by pioglitazone, confers efficacy in ADPKD treatment through multiple mechanisms. There is only one clinical trial (ongoing) wherein it is being assessed for its benefits and risk in patients with ADPKD, and is expected to get approval from the regulatory body owing to its promising therapeutic effects. This article would encompass the updated information on the epidemiology, pathophysiology of ADPKD, different mechanisms of action of pioglitazone in the treatment of ADPKD with preclinical and clinical shreds of evidence, and related safety updates.
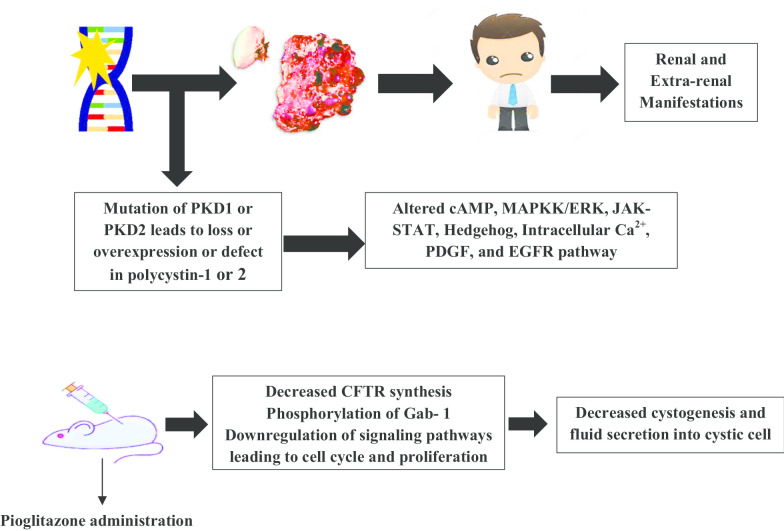

## Introduction

Autosomal dominant polycystic kidney disease (ADPKD) is an inherited chronic kidney disorder (CKD) that is characterized by the development of numerous fluid-filled cysts in kidneys (Bergmann et al. 2018). These cysts can arise in the form of outpouching from any part of the nephron and progressively destroy renal parenchyma. It is caused due to mutations either in the PKD1or PKD2 gene; the former is more common and accounts for 85% of the cases, while the latter accounts for the remaining cases (Bergmann et al. 2018; Cornec-Le et al. 2019). ADPKD is inherited dominantly, so, an individual will develop the cyst if one parent contains the mutated gene (Reddy and Chapman 2017). It impairs kidney function and progressively leads to end-stage renal disease (ESRD) in which a patient is needed to undergo dialysis or kidney transplant (Bergmann et al. 2018). This genetic disorder has affected around 12.5 million people worldwide regardless of sex and ethnicity and is the fourth leading cause for about 10% of patients with ESRD owing to which, it poses a considerable economic burden of approximately $10 billion (Nowak et al. 2018; Chapman et al. 2015). Although the exact mechanism behind cystogenesis is incompletely understood, a significant number of studies have attributed it to the alterations of different molecular signaling pathways that are regulated by genetic products of PKD1 and PKD2 that is polycystin-1 and polycystin-2, respectively found in primary cilia (Viau et al. 2020). ADPKD is incurable, and the treatment of ADPKD is limited to the management of different renal and extra-renal complications. The only approved medication to treat ADPKD is the tolvaptan (a vasopressin receptor-2 antagonist) that slows down the decline in kidney functions but is associated with a higher discontinuation rate owing to multiple adverse events (Bergmann et al. 2018; Torres et al. 2012). Pioglitazone; a well-known anti-diabetic drug that is a peroxisome proliferator-activated receptor gamma (PPAR-γ) agonist, has been found to suppress the development of renal cyst in the preclinical studies via different mechanisms which inarguably suggests its pleiotropy. The characteristic property to improve the molecular and phenotypic defects with a lower risk of adverse effects in preclinical animal models has proved pioglitazone an excellent and emerging candidate to treat ADPKD (Liu et al. 2020; Raphael et al. 2009a; Malas et al. 2020).

The first part of this review will encompass substantial information regarding the epidemiology, signaling pathways involved in the pathophysiology of ADPKD, and clinical manifestations of ADPKD. In the second part, different preclinical and clinical studies along with the mechanisms through which pioglitazone exerts beneficial pharmacological actions in ADPKD, and its safety perspectives will be reviewed.

## Epidemiology

Pierre Rayer first identified polycystic kidney disease in 1841 and Felix Lejars formally coined the term "polycystic kidney disease" in 1888 (Colbert et al. 2020). ADPKD is the most prevalent of all hereditary forms of CKD in the United States of America (USA), reaching 600,000–700,000. Across the globe, around 12.5 million people suffer from ADPKD and are responsible for about 10% of ESRD patients, and it equally affects both male and female (Nowak et al. 2018; Chapman et al. 2015). ADPKD is more common than hemophilia, sickle cell disease, cystic fibrosis, and Huntington disease combined (Masoumi et al. 2008). Every parent's offspring with ADPKD will inherit the mutant gene because it is completely penetrated (Harris and Torres 2020). The newly claimed incidence in the USA has been found to account for around 5000 cases/year which have increased with the advancement of technologies (Lanktree et al. 2018). It gradually progresses into the ESRD by the age of 60 in 50% of patients (Chebib and Torres 2016). It is attributed to the causation of 6% of cases of ESRD in the USA and has become the 4th leading cause of ESRD in adult patients in the European Union (Chebib and Torres 2016; Willey et al. 2017). Suwabe et al. reported the annual incidence of 3.06/100,000 patients in Olmsted County, Minnesota during 1980–2016, and the point prevalence was found to be 68/100,000 population on January 1, 2010 (Suwabe et al. 2020). Willey C et al. conducted a nationwide incidence and prevalence study of ADPKD in the USA for 3 years. The annual incidence and diagnosed prevalence were found to be 0.62/10,000 and 4.3/10,000 CKD patients, respectively (Willey et al. 2019). In India, there is a need for epidemiological studies for ADPKD as insignificant data is present that could rationally reflect its prevalence and incidence. Yersin et al*.* reported the prevalence data of ADPKD in the population of Seychelles (Indian Ocean). In this 3-year prospective study, the total prevalence was found to be 57/100,000 inhabitants. The age range of affected persons was found between 12 to 73 years with the mean age of 35.5 years, of which 25 patients were female, and 17 were male (Yersin et al. 1997).

## Clinical manifestations

### Pain

The very common complication which is found in 60% of individuals with ADPKD is Kidney pain. The pain can be either acute or chronic, depending upon the progression of the disease. Acute pain can be due to the cyst rupture, infection, and formation of kidney stones (Tellman et al. 2015). The unsynchronized and interrogated activities of autonomic and sensory neurons that innervate the kidneys are responsible for the chronic kidney pain in ADPKD (Casteleijn et al. 2014). Often, the management of pain involves analgesics, but the analgesia produced is not optimal. Hence, health care professionals have to rely upon other approaches like radiofrequency ablation, laparoscopic renal denervation, and spinal cord stimulation (Casteleijn et al. 2014; Tellman et al. 2015). Thoracoscopic sympathos planchnicectomy; another procedure that is used but it is quite invasive and has been seen to produce complications like orthostatic hypertension and pneumothorax (Casteleijn et al. 2014).

### Hypertension

It is a very common and early manifestation in patients with ADPKD, affecting around 60% of patients before the impairment in the kidney function (Chapman et al. 2010). The different studies have upheld different factors that are responsible for the increased BP in ADPKD, but the exact mechanism has not been shown in any of the studies (Helal et al. 2017; Rahbari-Oskoui et al. 2014; Ratnam and Nauli 2010). The two factors that are commonly sought are the activation of the sympathetic nervous system and renin–angiotensin–aldosterone system owing to the enlargement of the renal cyst (Rahbari-Oskoui et al. 2014). In a study, it was found that in hypertensive ADPKD patients, the renal volume is comparatively large as compared to the normotensive ADPKD patients (Grantham et al. 2006). The fact that hypertension is itself responsible for causing several cardiac complications cannot be ignored. Complications like left ventricular hypertrophy (LVH) is very troublesome and lead to morbidity and mortality in hypertensive ADPKD patients. Dad et al*.* reported that intensive BP control was found to significantly decrease the left ventricular mass index (LVMI) as measured by cardiac magnetic resonance imagining (MRI) in the hypertensive ADPKD patients (Harris and Rossetti 2010; Dad et al. 2018).

### Infertility and congenital anomaly

A very important aspect of APKD complication that affects a majority of males with a prevalence ranging from 39 to 60% (Joo et al. 2010; Luciano and Dahl 2014). The seminal vesicle cyst produced a tomographic attenuation with the values between 0 to 30 (Hounsfield unit) HU in a study by Alpern et al. due to an increase of thickness of seminal vesicle (3 to 4 cm). They reported that the defect in the basement membrane of the seminal vesicle could be responsible for the development of the cyst (Alpern et al. 1991).

Females are not directly affected by the ADPKD; their reproductive parts show no impairment, but the morbidity in ADPKD pregnant women is high as compared to the pregnant women with no ADPKD (Bergmann 2015). Furthermore, there is an increased rate (28%) of fetal prematurity in ADPKD preeclamptic women as compared to normotensive ADPKD women (10%). The normotensive women have uncomplicated pregnancies and fewer odds of fetal complications (McBride et al. 2020).

### Abdominal hernia

This is another very troublesome extra-renal complication of ADPKD that is very prevalent (Dupont et al. 2018). A study involving the comparison of renal failure patients with and without ADPKD found the abdominal hernia prevalence of 45% (38/85) and 8% (7/85) in patients with renal failure with and without ADPKD, respectively (Mikolajczyk et al. 2017). Furthermore, Banshodani et al. in their study reported that there was a significant number of incisional (P = 0.019), inguinal (P < 0.001), and para-umbilical hernia (P = 0.007) in ADPKD patients as compared to their counterpart group (Morris-Stiff et al. 1997).

### Liver cyst

Another frequent complication of ADPKD that cannot be left unreviewed. The prevalence of liver cyst in ADPKD can be understood from the study of Bae et al. who found that the overall prevalence of hepatic cyst in 230 ADPKD patients was 83% of which 85% women, and 79% men had a liver cyst. They also found that prevalence was directly related to renal cyst volume and renal volume. The volume of the hepatic cyst was sequentially related to the age of the patient; with 0.25 ml and 22.78 ml for the age group of 15 to 24 y and 35 to 46 y, respectively (Bae et al. 2006). Though both males and females have a lifetime risk for hepatic cyst, but the number and size of hepatic cysts are found to be greater in females than males. Gabow PA et al*.* in their study reported that females were more likely to develop the numerous hepatic cysts than males; females had > 15 hepatic cysts as compared to males (P < 0.04). The mean size of the hepatic cyst was higher in women (4.2 ± 0.4 cm) than in men (2.7 ± 0.3 cm). There was a striking correlation between the pregnancy and hepatic cysts; a total of 90% of women with ADPKD and liver cysts had been pregnant as compared to non-pregnant women with ADPKD and liver cyst (63%). Furthermore, the hepatic cystic size was bigger who had been pregnant than non-pregnant women (4.5 ± 0.4 cm vs 1.7 ± 0.5 cm). The female steroid hormones were sought to be the responsible factor that may regulate the growth of biliary epithelium. The changes at the cellular level pathogenesis indicate that cyst originates from biliary microhamartomas, which represents the overgrowth of bile ducts (Gabow et al. 1990).

### Aneurysm

This extra-renal vascular complication due to ADPKD is a unique fearful complication among all the above-mentioned. The real trouble occurs when the aneurysm ruptures and leads to internal bleed and mortality (Jung 2018). Sanchis et al. reported in their observational study that there was a total of 94 intracranial aneurysms in 75 of 812 ADPKD patients. Most of the diagnosed aneurysm was small in size with a median diameter of 4 mm. Furthermore, hypertension and smoking were found to be very common in patients with aneurysm (Sanchis et al. 2019). Lozano-Vilardell et al. reported a case of an infrarenal abdominal aortic aneurysm (AAA) in a patient with ADPKD. In their study, they mentioned that AAA had a short neck, and performing the standard endovascular aortic repair did not seem to be worthy. The patient underwent embolization of the two renal arteries with coil, and the endoprosthesis had to be carried out to the level of the superior mesenteric artery. Fortunately, the patient's condition improved and showed no further complications (Lozano-Vilardell et al. 2019). The underlying mechanism for an aneurysm in the ADPKD patient has been attributed to the PKD1 and PKD2 gene that is also expressed in the vascular smooth muscle wall that gets mutated in the ADPKD. The wall helps to keep the cell–matrix and cell–cell mechanical coupling among the several layers of tunica media (Silverio et al. 2015).

## Pathophysiology

### Polycystin-1 and polycystin-2: structure and role

To understand the pathogenesis of ADPKD, it is important to emphasize the products of PKD1 (present on chromosome 16p13.3) and PKD2 (present on chromosome 4q21) genes. PKD1 and PKD2 encode polycystin-1 and polycystin-2 protein, respectively. The mutation in the PKD1 gene is the leading factor behind 86% of cases of ADPKD, while the remaining 15% of ADPKD patients have the PKD2 mutation (Cornec-Le et al. 2019; Hafizi et al. 2014). The former groups of patients are found comparatively with a greater number of renal cysts and progresses rapidly to ESRD (Cornec-Le et al. 2019). The structure of polycystin-1 enables it to perform a specific function. Decoding the polycystin-1, we find that it comprises 4303 amino acids with 11 transmembrane domains, a small cytoplasmic terminal (C-terminus) that interacts with different proteins including the polycystin-2 (Inoue et al. 2014). The long extracellular terminal (N-terminus) interacts with molecules like proteins, fat, and carbohydrate and receives the signals through which the cell responds to its environment. Polycystin-1 is found in bone, kidney, brain, and muscle (Piperi and Basdra 2015). In the kidney, polycystin-1 in the kidney is particularly found in the distal nephron and collecting duct, but more recently it was located in the primary cilia of epithelial cells of the collecting duct. Primary cilia are the sensory organelle that bugles outside the lumen (Lee and Somlo 2014). Polycystin-2, unlike polycystin-1, is comparatively smaller with six transmembrane domains and 968 amino acids (Giamarchi et al. 2010). It is mainly a calcium-permeable ion channel that is found in the endoplasmic reticulum (ER) membrane and regulates the release of calcium ions from the ER, and cell proliferation and differentiation. Polycystin-2 is also located at the basolateral cell membrane of tubular epithelial cells containing the primary cilia of the kidney (Vien et al. 2020).

### Molecular pathways

While the exact pathological roles of these proteins are still debated but it is clear that cystogenesis in ADPKD takes place when both the copies of either PKD 1 or PKD2 gets mutated and cause the defect in the primary cilia owing to the change in the genetic expression of aforementioned genes (Cornec-Le et al. 2019; Harris 2010). The resulting abrupt expression (under-expression or over-expression) of polycystin-1/2 leads to the disruption of several intracellular signaling pathways that further lead to the progressive development of cyst due to dysregulation of cell proliferation and fluid secretion into the cyst (Harris 2010; Paul and Vanden Heuvel 2014). The next section is centered on reviewing the most commonly sought polycystin-1/2 mediated molecular signaling pathways that are found to be affected in ADPKD.

### Cyclic AMP pathway

Cyclic adenosine monophosphate (cAMP) is one of the key components in renal cystic growth which is quite evident from the study of Yamaguchi et al. who investigated the effect of cAMP on the in vitro cystic cell proliferation derived from ADPKD cyst of human. 8-Br-cAMP in the concentration of 100 µmol/L was found to stimulate cell proliferation (99% above the baseline) from eight different subjects with ADPKD. The adenylate cyclase agonist; forskolin (10 µmol/L) was found to increase the proliferation to 124%. The cAMP-dependent protein kinase, when inhibited by Rp-cAMP and H-89, inhibited the proliferation of cystic cells. In the same study, they concluded that cAMP agonists induce the proliferation in ADPKD through the protein kinase activation of the extracellular signal-regulated kinase (ERK) pathway (Yamaguchi et al. 2000). Now, how the polycystin-1 relates to the development of ADPKD can be sought from the study of Sutters et al. who conducted their study to find the effect of overexpression of C-terminal of polycystin-1 on the cAMP-responsive cell proliferation of M-1 cells (a cell that retains the characteristic of cells of collecting duct). They found that the over-expression of the C-terminal of polycystin-1 abrupted the normal cellular signaling and transformed the normal M-1 cells into an abnormal PKD cell (Sutters et al. 2001). Hanaoka et al. reported the effect of cAMP on the proliferation of ADPKD epithelial cell and cyst growth and normal kidney cortex cells. They found that cAMP-induced the deoxyribonucleic acid (DNA) synthesis in ADPKD epithelium and led to cell proliferation, but the effect was not found on the normal kidney cortical cells. Apart from the above-mentioned, they also found that cAMP stimulated the enlargement of cystic cells and there was an additive effect on the cell growth when cells of ADPKD were treated with cAMP and epidermal growth factor (Hanaoka and Guggino 2000).

The review of the cAMP role in ADPKD will be incomplete without discussing the tolvaptan, and vasopressin [also called arginine vasopressin (AVP)] in the context of ADPKD. Tolvaptan is a non-peptide, selective vasopressin type-2 receptor (V2R) antagonist. V2R is primarily expressed on the cells of the collecting duct of the nephron and promotes the reabsorption of water (Boone and Deen 2008). The role of vasopressin in ADPKD is substantial and has been proved in different studies. A V2R agonist, in an animal model, was found to increase the cAMP level and hence, aggravated the cystogenesis but the same got decreased when tolvaptan was administered. Wang et al*.* reported that in PCK^−/−^-AVP^−/−^ rat, which was formed by crossing the PKD1 deficient; PCK^−/−^, and Brattleboro AVP^−/−^ rat, treatment with 1-deamino-8-d-arginine vasopressin (V2R agonist) increased the level of renal cAMP and had an increased cyst volume. But, when they were administered with tolvaptan, these rats had a lower cAMP level, and a lowered cystogenesis marked by an increase in the renal mass without any induction of cystic growth which suggested the protective role of tolvaptan in ADPKD (Wang et al. 2008).

### Hedgehog pathway

**Fig. 1:**
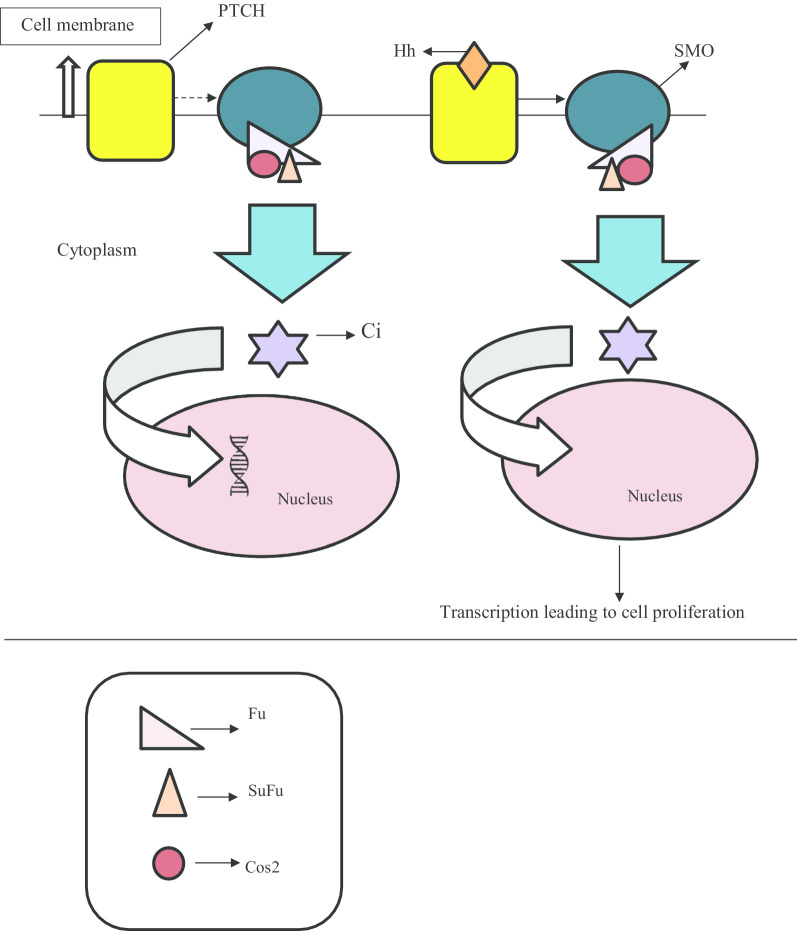
Illustration of the hedgehog pathway. Hh: Hedgehog; SMO: Smoothened; PTCH: Patched; SuFu: Suppressor of Fused; Cos 2: Kinesin-like molecule costal 2; Ci: Cubitus interruptus; Fu: Serine/threonine protein kinase Fused. In the absence of the Hh ligand, PTCH receptor suppresses (shown by the dashed line) the G-protein coupled receptor (GPCR) named SMO. Hence, no further signaling cascade takes place which prevents cell proliferation. Hh signaling gets activated when a Hh ligand such as Sonic Hh binds to the PTCH. This ligand binding relieves the SMO, thereby it to modulate a complex known as hedgehog signaling complex (HSC). HSC is comprised of four different proteins namely SuFu, Fu, Cos2, and Ci. Here, in the figure, Ci is not shown in association with HSC. In the absence of Hh ligand, Cos2-Fu-Ci complex interacts with the C-tail of SMO which leads to the active form of Ci (Ci-A) that is responsible for the activation of target genes, leading to cellular processes like cell proliferation, and differentiation. In the absence of the Hh ligand, SuFu-Ci and Cos2-Fu-Ci complexes promote the repressor form of Ci (Ci-R), thereby preventing it to activate the target genes (Jia et al. 2015)

The Hedgehog (Hh) signaling regulates cell proliferation, differentiation, and tissue polarity in the primary cilia. The hedgehog signaling is started when the hedgehog ligand like Desert/Indian/Sonic Hh binds to the transmembrane receptor called Patched (PTCH). When there is no ligand, PTCH represses the activation of a G-protein-coupled receptor called Smoothened (SMO). Upon binding of extracellular Hh ligand, PTCH gets relieved, allowing SMO to modulate a cytoplasmic complex known as the Hh signaling complex (HSC). This complex targets a homolog of glioma-associated (GLI) transcription factor called Cubitus interruptus (Ci), leading to its proteolytic cleavage. The Ci is the major downstream component of Hh signaling that, depending upon the Hh stimulation, can function both as a transcriptional activator or repressor of target genes involved in the cellular processes like cell proliferation (Jia et al. 2015) (Fig. 1). There are a significant number of studies that certify the involvement of Hh signaling in ADPKD. Silva et al. reported that Hedgehog inhibitors; Sant 2 (SMO antagonist) and Gant61 (GLI inhibitor), reduced the cell proliferation in ADPKD cells (Ogden et al. 2004). Tran et al*.* reported that cAMP-mediated cystogenic potential of tetratricopeptide repeat-containing hedgehog modulator-1 (THM1); a protein whose deficiency has been found to cause the renal cysts, got reduced when GLI2 (a transcription activator of Hh pathway) was genetically deleted in a mouse model of ADPKD. They also found that when the THM1 conditional knockout mice (THM1 cko) and GLI2^−/−^ mice were treated with Gant61 (a GLI antagonist) in the presence of 8-Br-cAMP; milder cystic growth was seen in (THM1 cko + GLI2^−/−^) mice as compared to THM1 cko, thus suggesting the role of Hh pathway in renal cystogenesis in ADPKD (Tran et al. 2014).

A study conducted by Ma et al*.* created an ambiguity in the certainty of the role of Hh signaling in the formation of cysts in ADPKD. The observations of this study contradicted the above researches. They reported that inactivation of the hedgehog pathway in a PKD1^Cre^; SMO^fl/fl^ mice, in which there is the deletion of PKD1 and SMO genes, led to strong cystic phenotypes. Next, they investigated the effect of Hh signaling on the cyst by using the animal model; PKD1^Cre^; ROSALSL^−GFP−SMO−M2^ mice that expressed GFP-fused form of SMO along with deleted PKD1. This concomitant inactivation of PKD1 and expression of SMO-M2 did not induce any cystic growth (Ma et al. 2019).

### Intracellular calcium

**Fig. 2:**
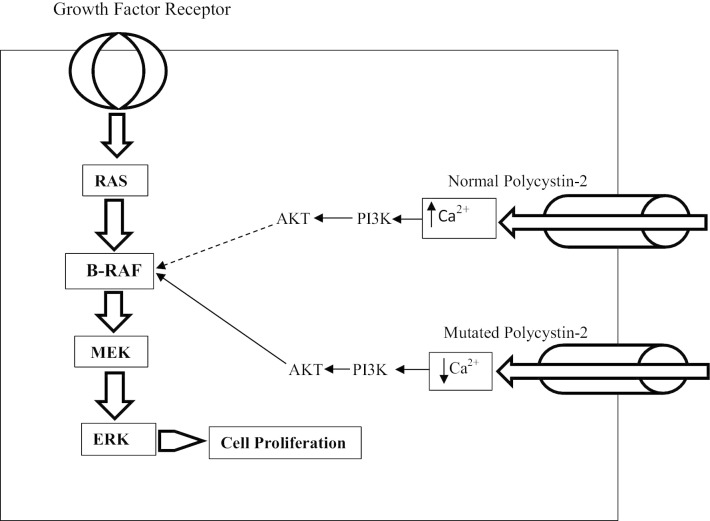
Pictorial representation of the role of intracellular calcium in the pathophysiology of ADPKD. ADPKD: Autosomal dominant polycystic kidney disease; PI3K: Phosphatidylinositol-3-kinase. In a normal kidney epithelial cell, there is an increased level of intracellular calcium due to the proper functioning of polycystin-2 at the basolateral membrane and endoplasmic reticulum (not shown in the figure) along with other calcium ion channels. Increased calcium leads to the activation of PI3K that further activates the Akt. Akt leads to the inhibition of the proliferative factor; B-Raf and therefore abate the activation of other factors of Ras/Raf/MEK/ERK pathway (shown by the dashed line). In a renal epithelial cell with mutated polycystin-2, there is a low intracellular calcium level due to which B-Raf cannot get inhibited by the decreased formation of Akt that consequently leads to unabated cell proliferation (Mitobe et al. 2010; Li et al. 2016)

The primary cilia are the non-motile organelles that bulge from the apical side of the epithelial cells to the outside of the lumen of the nephron (Marra et al. 2016). In the kidney, they function as mechanical and chemo-sensors; translating the extracellular stimuli into the calcium ion influx through polycystin-2 (Sutters et al. 2001). This influx induces intracellular calcium release from ER that regulates various processes like cell proliferation, differentiation, gene expression, and apoptosis (Sutters et al. 2001; Mekahli et al. 2011). It is the notion in different studies that cells from the kidney of ADPKD show a reduced level of intracellular calcium and an increased cAMP. Mise and their colleagues conducted a study to evaluate the role of activation of the calcium-sensitive receptor in PKD1 deficient cells. They reported that the NPS-R568 (an activator of calcium channel receptor) in a conditionally immortalized proximal tubular epithelial cells (ciPTEC) derived from a patient with ADPKD showed an increased calcium level and reduced activity of cAMP and mammalian target of rapamycin (mTOR) as compared to its wild type (wt) clone; ciPTECwt, that indicated the potential role of intracellular calcium in ADPKD (Mise et al. 2018). Furthermore, several preclinical studies have reported that calcium channel blockers aggravated the ADPKD that indicates that low calcium level acts as a proliferative factor in epithelial cells of cystic kidneys (Nagao et al. 2008; Mitobe et al. 2010). In a normal kidney epithelial cell, intracellular calcium leads to the activation of phosphatidylinositol-3-kinase (PI3K) that in turn leads to the activation of protein kinase B (Akt). B-Raf protein (a type of Raf protein), which can activate the MEK of Ras/Raf/MEK/ERK pathway, gets inhibited by Akt. Due to the defect in polycystin-2 (calcium channel) in ADPKD, there is a decreased intracellular calcium and this could not activate the PI3K, therefore an unfortunate activation of Raf takes place that ultimately is accountable for the cell proliferation via Ras/Raf/MEK/ERK pathway (Li et al. 2016). The illustration of the role of intracellular calcium and polycystin-2 in ADPKD is shown in Fig. 2.

### Cystic fibrosis transmembrane conductance regulator (CFTR)

The key characteristic of ADPKD is the development of a large number of the fluid-filled cyst, which is mainly based on CFTR (Mangolini et al. 2016). CFTR is a chloride ion channel that is encoded by the CFTR gene whose mutation has been linked to be responsible for the fluid secretion by the thin-walled epithelium of cyst. It is found in the apical membrane of the epithelial cells of the liver, and proximal and distal tubule of the kidney (Sun et al. 2011; Saint-Criq and Gray 2017). Different studies have confirmed that to maintain the integrity of cyst, trans-tubular secretion is required which is facilitated by chloride secretion through the CFTR ion channel that acts as an electrochemical driving force. The study of Hanaoka et al. is one such study aimed to find the role of CFTR in the pathophysiology of ADPKD by immunocytochemical and patch-clamp technique. They reported that cAMP activated Cl^−^ currents were found in cultured cells of ADPKD. Furthermore, the currents had characteristics of linear current–voltage relation, sensitivity to anion in the sequence of Br- > Cl^−^ and sensitivity to diphenylamine, and glibenclamide, all of which were identical to CFTR (Verkman et al. 2013). In the review study of Li et al., the therapeutic potential of CFTR inhibitors in ADPKD is probed. The small molecules like thiazolidinedione CFTR (inh)-172 and GlyH-101, regarded as the novel CFTR inhibitors, have been found to inhibit the fluid secretion in cyst (Hanaoka et al. 1996).

### JAK/STAT pathway

**Fig. 3:**
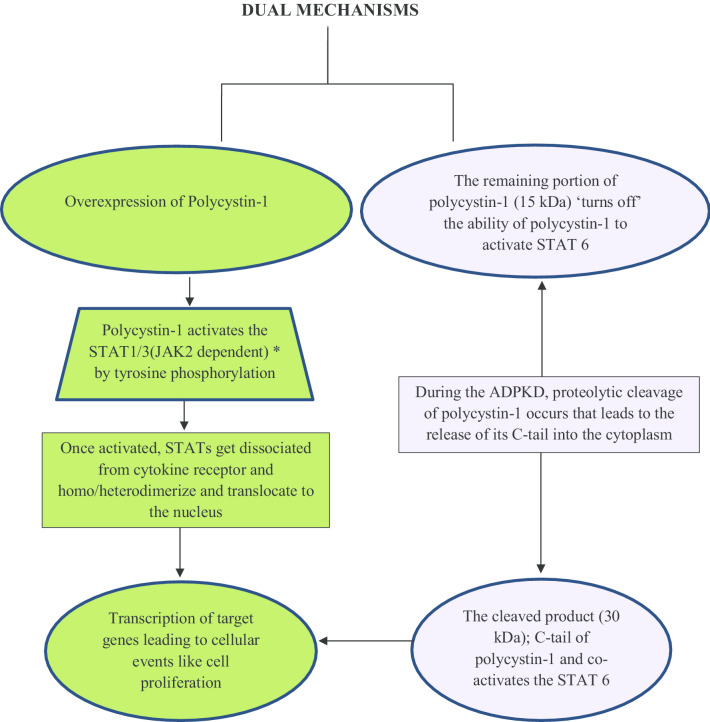
The mechanism of STAT signaling by Polycystin-1. In autosomal dominant polycystic kidney disease (ADPKD), mutation of the PKD1 can lead to either the reduced or overexpression of polycystin-1. The over-expression of polycystin-1 (membrane-anchored) stimulates the activation of STAT1/3 by its phosphorylation of tyrosine. *Polycystin-1 binds to JAK2 that suggests polycystin-1 mediated regulation of JAK2 is attributable to the STAT1 activation. The other half of the figure is showing another model adopting which polycystin-1 regulates STAT proteins. In the ADPKD state, the 30 kilodalton (kDa) cytoplasmic (C) tail of polycystin-1 is released into the cytoplasm after cleavage and translocate to the nucleus where it interacts with the transcriptional co-activator P100 and STAT6 and co activates the STAT6. The remaining membrane-anchored portion (15 kDa) of polycystin-1 inhibits the STAT6 as it loses the ability to activate STAT6 (Ma et al. 2005)

Janus activated kinase (JAK)-Signal transducer and activator of transcription (STAT) signaling pathway is known to mediate the cellular response to different growth factors and cytokines (Seif et al. 2017). The renal expression of JAK and STAT are abnormally high in ADPKD that is in line with the findings of different studies (Patera et al. 2019; Fragiadaki et al. 2017). Bhunia et al. reported that embryo of the mouse who lacked the PKD1 had defective phosphorylation of STAT1 and p21^waf^ induction. P21^waf^ is one of the cyclin-dependent kinase inhibitors whose expression level was found to be high in cells overexpressing the polycystin-1 (Bhunia et al. 2002). Patera et al. investigated the role of JAK2 in ADPKD in the mice model (Pkd1nl/nl). They found that there was the overexpression of JAK2 lining the cystic cells in ADPKD when compared to the normal kidneys. The expression was seen more ectopically in the interstitial. The inhibitors of JAK2, namely curcumin and tofacitinib, showed reduced growth of cyst; that indicated the potential role of the JAK pathway in ADPKD (Torres et al. 2012). The mechanistic pathway through which polycystin-1 leads to cellular proliferation by regulating the JAK-STAT activity is shown in Fig. 3. There is a dual mechanism through which polycystin-1 regulates the JAK-STAT activity in ADPKD. There is clear evidence that ADPKD not only involves the reduced expression of polycystin-1 due to the mutation in PKD1, but it also involves the polycystin-1 overexpression. Using the aforementioned phenomenon, it abrupts the JAK-STAT signaling and leads to the cellular proliferation of kidney epithelial cells (Weimbs et al. 2013).

### EGFR pathway

**Fig. 4:**
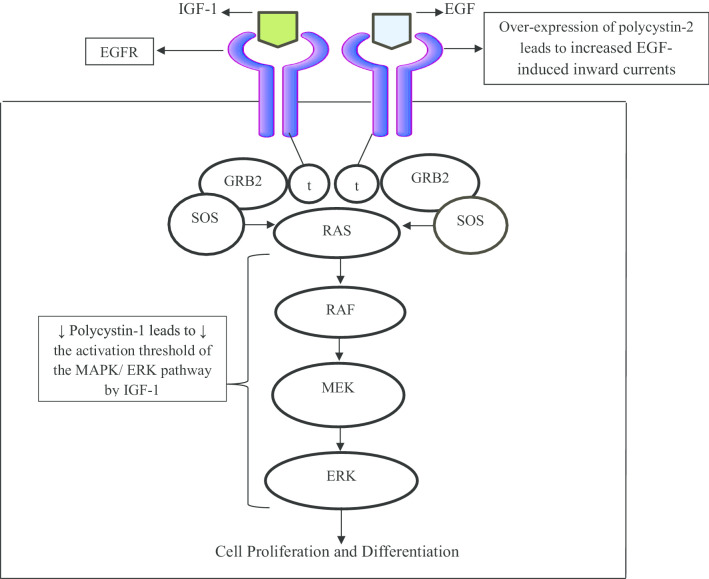
Illustration of the effects of polycystins dysfunctioning on EGFR and MAPK/ERK pathway. "Epidermal growth factor" (EGF) and "Insulin-like growth factor-1" (IGF-1) act as ligands for the epidermal growth factor receptor (EGFR). When gets activated by these ligands, dimerization of receptor is induced that leads to the activation of the tyrosine kinase activity of the receptor which further leads to the phosphorylation of tyrosine residues on each other (autophosphorylation) to form phosphotyrosine (shown by letter, 't'). The “growth factor receptor-bound protein-2” (GRB2) is an adaptor protein that helps to transduce the signals from EGFR to "Rouss avian sarcoma" (RAS) protein. GRB2 binds to phosphotyrosine and SOS protein via its SH2 and SH3 domains, respectively. The GRB2/SOS complex now activates the RAS. RAS in turn activates the RAF (protein kinase activity of RAF gets activated) that phosphorylates the mitogen-activated protein kinase kinase (MEK). MEK phosphorylates the extracellular signal-regulated kinase (ERK); also known as mitogen-activated protein kinase (MAPK) which further phosphorylates several other proteins that regulate cell proliferation, and differentiation. Decreased polycystin-1 expression lowers the activation threshold of the MAPK/ ERK pathway by IGF-1 and increased EGF-induced inward currents in kidney epithelial cell lines are produced due to over-expression of polycystin-2; together lead cystogenesis (Ma et al. 2005; Parker et al. 2007)

In mammalian cells, this pathway plays a very important role in cell growth, proliferation, and differentiation (Wee and Wang 2017). There is an augmented activity of epidermal growth factor receptor (EGFR) in the kidneys of patients with ADPKD. Particularly in this regard, the study of Du et al. holds great significance. They reported that > 1 ng/ml of EGF induced mitogenic activity in ADPKD epithelium. Additionally, the fluid from the early and end-stage ADPKD showed a mean of 2.8 ng/ml and 1.4 ng/ml EGF, respectively. They found a high-affinity binding of EGF to the EGFR to the apical membrane of ADPKD, but the same could not be found in normal kidneys (Du and Wilson 1995). Another landmark study by Ma et al. showed that over-expression of polycystin-2 increased the EGF-induced inward currents in kidney epithelial cell lines; LLC-PK1. Taking the findings together, it can be said that EGF plays a very important role in ADPKD cell proliferation (Ma et al. 2005). Fig. 4 shows the effect of malfunctioning of polycystin-1 and 2 on the EGFR pathway.

### RAS/RAF/MEK/ERK and PDGF pathway

Yamaguchi et al. reported the effect of EGF on the activation of RAS/RAF/MEK/ERK pathway, also known as MAPK/ERK pathway and they found that EGF stimulated the phosphorylation of Raf and ERK in primary cultures made of human ADPKD but could not stimulate the phosphorylation in normal kidney cell. The activation of the RAS/RAF signaling pathway in ADPKD is attributed to a decreased number of polycystin-1 (Yamaguchi et al. 2010). Parker et al. reported that a decrease in the number of polycystin-1 or insufficiency leads to a reduced activation threshold of the MAPK/ERK signaling pathway that ultimately leads to the growth factors like “insulin-like growth factor-1” (IGF-1) induced hyperproliferation of tubular epithelial cells (Parker et al. 2007) (Fig. 4). Another growth factor; platelet-derived growth factor (PDGF)'s mRNA expression level was found to be highly increased with the advancement of cyst in the DBA/2FG-pcy mice. These mice have very similar properties that are seen in the kidneys of patients with ADPKD. The researchers reported that mRNA expression levels of PDGF-A and PDGF-B at 30 weeks of age were increased to 3.7-fold and 4.6-fold, respectively in DBA/2FG-pcy mice that indicate a significant role of PDFG in the progression of cystic kidney disease (Nakamura et al. 1993).

## Mechanism of action

Pioglitazone belongs to the family of thiazolidinedione that confers its therapeutic effect through the activation of PPAR-γ that is expressed in different tissues of the body, including the kidney. These receptors belong to the category of nuclear receptor superfamily. PPAR-γ associates with the retinoid X receptor to form the heterodimer. This heterodimer binds to the promoter region of specific DNA sequences (peroxisome proliferator response elements) of target genes and modulates their transcription (Compendium and Actos Tablets 2020; Iglesias and Díez 2006). PPAR-γ have been further subdivided into four types, namely γ1, γ2, γ3, γ4 that are present in different organs and tissues. The activation of PPAR-gamma leads to various actions that consequently leads to the actions that include transactivation of the genes that regulate the adipocyte differentiation that leads to an increased number of small insulin-sensitive adipocytes, increased uptake of glucose by skeletal muscle, and decreased production of glucose by the liver which consequently are responsible for its glucose-lowering effect in type-2 diabetes mellitus patients; thereby decreasing the insulin resistance (Iglesias and Díez 2006; Tyagi et al. 2011). It is well known that tumor necrosis factor-α (TNF-α) is responsible for causing insulin resistance, but the administration of pioglitazone has shown to reduce the expression of TNF-α. Due to all of the above factors, it is popularly called as an insulin sensitizer (Quinn et al. 2008). Through PPAR-γ activation, pioglitazone exerts beneficial actions in ADPKD, which is discussed in the next section.

## Shreds of evidence proving the efficacy of pioglitazone

### Preclinical evidence

Blazer-Yost et al. conducted a study to find the efficacy of pioglitazone in ADPKD by using the PCK rat model, which progressively develops cystic enlargement of the kidneys. They observed a substantial decrease in the cyst burden in male rats at a dose of 20 mg/kg of body weight after a 7-week time duration. The mean renal cystic volume was found to be lower in female rats for the fed with pioglitazone as compared to the control group (0.31 ± 0.09 ml vs 0.39 ± 0.05 ml), respectively. A statistically significant difference (P = 0.04) was seen between the pioglitazone treated (0.31 ± 0.04 ml) and control group (0.72 ± 0.06 ml). After 14-weeks of administration of pioglitazone, in the group of female rats, renal cystic volume was 0.42 ± 0.09 ml while in the control group, it was 0.66 ± 0.06 ml with a statistically significant difference of P = 0.035. The effect of pioglitazone on the development of cyst was also tested at the dose of 4 mg/kg bodyweight for 7 weeks, and they found that this dose was as effective as the higher dose (20 mg/kg) in lowering the cystic burden in both the male and female rats. In both cases, a statistically significant difference between both the male (P = 0.006) and female (P = 0.016) animals of control, and the pioglitazone group was found. The mean renal cystic volume of female rats in the control and pioglitazone group was 0.39 ± 0.05 ml and 0.30 ± 0.03 ml, respectively while in male rats it was 0.71 ± 0.06 ml and 0.50 ± 0.03 ml, respectively. In this study, the mechanism of action of pioglitazone for this pharmacological action was attributed to the decrease in the CFTR ion channel synthesis, which consequently decreased the fluid secretion in the cystic epithelium. The authors found that there was CFTR-positive staining in the apical membrane lining the liver cysts in the rats with ADPKD while it was much diminished in the rats treated with pioglitazone (P = 0.009) (Blazer-Yost et al. 2010).

Yoshihara et al. performed a study to rule out the effect of pioglitazone on the expression of genes in the PCK rat model of human ADPKD. The genes of several pathways were down-regulated when the mice were administered with 10 mg/kg pioglitazone for 20 weeks. The Gene Set Enrichment Analysis (GSEA) showed that those gene sets were down-regulated in pioglitazone treated animals that were related to regulating cell proliferation and cell cycle pathways; JNK, PDGF, and EGF. The gene set that is involved in the regulation of inflammatory signals, namely interleukin-1 and interleukin-6 pathways, were too down-regulated (Yoshihara et al. 2012). Stearoyl-coenzyme desaturase 1 (SCD1) gene stimulates cell proliferation in the cancer cells with the help of a growth factor; protein kinase B, which is one of the kinases responsible for cystic cell proliferation in PKD (Bhaskar et al. 2015). The Kyoto Encyclopedia of Genes and Genomes (KEGG) analysis showed that SCD1 was down-regulated in pioglitazone treated animals with PKD (Yoshihara et al. 2012).

Zhou et al. reported that JNK level was high in cystic epithelial cells in the PKD1 knockout mice; JNK caused the apoptosis of PKD1^+/+^ epithelial cells of renal tubule that indicates that the JNK pathway is involved to play roles in cell apoptosis (Zhou and Li 2015). The down-regulation of the JNK pathway in the study of Yoshihara et al. certifies the mechanism for the beneficial action of pioglitazone in ADPKD (Yoshihara et al. 2012).

Flaig et al. in their research have reported the efficacy of two PPAR-gamma agonists that is pioglitazone and rosiglitazone in the treatment of PKD by using the two rodent models, namely PCK and Wistar polycystic kidney (WPK^−/−^) rat model. WPK rat has a mutation in the TMEM67 gene and shows a rapid progressive cystic disorder. The most interesting thing was that the dosing of the two PPAR-gamma agonists was based on the reference of the equivalent dose that is used in humans. The pharmacological and sub-pharmacological doses of pioglitazone were 2.0 mg/kg and 0.2 mg/kg body weight in the WPK^−/−^ model, and for rosiglitazone, it was 0.4 mg/kg and 0.04 mg/kg body weight in the PCK model. In both the models, a statistically significant decrease in renal cyst burden (measured by total kidney weight) was seen with the sub-pharmacological doses of drugs. It is well established that the adverse drug effects related to these drugs are dose-related and therefore it will not be wrong to state that both the pioglitazone and rosiglitazone at a low dose will confer the benefit with lower risk which is evident from the above study. The authors of this study did not mention the exact mechanism of pioglitazone for this reduction in the cyst size; however, they postulated that it could be because of the inhibitory effect on the expression of apical CFTR in the kidney because of pioglitazone agonistic effect on PPAR-γ (Flaig et al. 2016).

Hypertension is a very common symptom in people with ADPKD and plays a very important role in the progression of cysts, as evidenced by different studies. Furthermore, these studies have very clearly described that there is the worst renal function of patients with PKD with hypertension (Helal et al. 2017; Grantham et al. 2006). A study of Raphael et al. showed an increase in the survival of homozygous PKD1 mutant animal model: PC-PKD1-KO (where PC and KO stand for Polycystic and Knock-out, respectively) mice when they were administered with pioglitazone (30 mg/kg). For the generation of PC-PKD1-KO mice, female mice heterozygous for PKD1^*cond*^ allele with Cre-recombinase expression under the control of aquaporin-2 promoter (APQ-2 Cre) were bred with male mice who were homozygous for PKD1 allele. The classical property of PKD1^*cond*^ mice is that they have the LoxP flanked exons 1 and 4 of the PKD1. The mean survival was found to be 57.2 ± 4.9 days in the control animals, but it was 90.2 ± 11.5 days in the animals who received pioglitazone (P = 0.034). This increase of survival was attributed to the blood pressure-lowering effect of pioglitazone; PC-PKd1-KO mice were hypertensive, but when treated with pioglitazone, the systolic blood pressure got reduced from 163.2 ± 11.7 to 128.2 ± 3.5 mm Hg. Though the mechanisms through which pioglitazone reduced the blood pressure was unclear, however, authors suggested that it could be due to the improved endothelial action and/or reduction of inflammation through which pioglitazone lowered the blood pressure (Raphael et al. 2009b).

Kanhai et al*.* investigated the efficacy of tolvaptan, pioglitazone, and combinational effect of tolvaptan and pioglitazone in a PKD^−/−^ preclinical model. They generated the (tam-KspCad-CreER^*T2*^ *(Cre);* Pkd1^*del2−1 l/lox2−11*^) mice lines in which exons 2–11 of PKD1 were either deleted (PKD1^*del2–*11^) or were flanked by LoxP (PKD1^lox2–11^). The in-vitro 3-D assay of pioglitazone showed that pioglitazone dose-dependently inhibited cyst swelling in cultured Pkd1^−/−^ cells; with the minimum, and maximum effect at 0.1 µM and 100 µM, respectively. Further, in this study, they reported that tolvaptan (0.15%) alone improved renal survival, decreased the cystic index (percent of cyst occupying the kidney), and blood urea nitrogen (BUN) level. Surprisingly, pioglitazone alone did not have a beneficial effect on these parameters, but the combinational therapy of tolvaptan and pioglitazone did show a better renal survival efficiency; though this difference was not statistically significant (P = 0.1325). The above-mentioned parameters were similar between the tolvaptan, and combination group (Kanhai et al. 2020).

Another study which was conducted by Muto et al. found the improvements in the molecular defects arising out of the deletion of the PKD1 gene in mouse embryos when the pioglitazone was administered. For the generation of PKD1^−/−^ mice, they targeted the disruption of exons 2–6 of PKD1 with the help of an MC1 promoter-driven neo-resistant gene that acted as a PKD1 targeting vector. Two embryonic stem cells (ESCs) were injected into the C57BL/6 J blastocysts, and germline transmission was achieved to produce the heterozygous PKD^±^ mice. Finally, these heterozygous mutants were crossed to produce the homozygous PKD1^−/−^ mice. The loss of polycystin-1 owing to the PKD1 deletion led to a decrease in the total protein level of beta-catenin in the heart and kidney, and c-MYC in the heart. The kidney expression of E-cadherin and platelet endothelial cell adhesion molecule-1 (PECAM-1) attenuated in the basolateral membrane of the renal tubule. When a dose of pioglitazone (80 mg/kg/day) was administered during the embryonic period, the stability of B-catenin and C-MYC were found to be increased. The cardiac double outlet of the right ventricle (DORV) was not found in the treatment groups, but it got found in the control groups. The expression of E-cadherin and PECAM-1 was increased to normal following the pioglitazone treatment. The increased tyrosine phosphorylation of EGFR and GAB1 in untreated PKD1^−/−^ got reduced after the pioglitazone treatment. The mechanism of action of pioglitazone conferring the improvements in the molecular defects remains unclear. However, the authors mentioned the involvement of cystogenesis inhibition via the quenching of phosphorylation of EGFR and GAB1 (Muto et al. 2002).

A summary of all the preclinical studies related to the effect of pioglitazone on ADPKD is shown in Table 1.

**Table 1:** Different preclinical studies evidencing the efficacy of pioglitazone in ADPKD

Primary citation of study	Preclinical model	^^Dose of pioglitazone	Duration of treatment	Observation	Mechanism
Yoshihara et al. (2012)	PCK rat	10 mg/kg	16 weeks	Decreased cell proliferation and reduced PKD progression	Gene sets responsible for cell cycle and proliferation were downregulated
Raphael et al. (2009)	PC-PKD1-KO mice	30 mg/kg	3 weeks	Pioglitazone increased the survival	^Blood pressure reduction due to improved endothelial function and/or reduction of inflammation
Blazer-Yost et al. (2010)	PCK rat	4 mg/kg and 20 mg/kg	7 and 14 weeks	With 20 mg/kg cystic burden^¥^ reduced after 7 weeks in male rats and 14 weeks in female rats and 4 mg/kg was effective in both the sexes after 7 weeks	A decrease in the synthesis of CFTR ion channel
Muto et al. (2002)	PKD1^−/−^ mice embryo	80 mg/kg	Embryonic day (7–11)	Decreased cardiac defects and renal cystogenesis	^Tyrosine phosphorylation of GAB1 and EGFR
Flaig et al. (2016)	WPK^−/−^ rat	2 and 0.2 mg/kg	13 days	Inhibition of cyst growth by 0.2 mg/kg	^Decrease in the CFTR expression
Kanhai et al. (2020)	PKD mice	30 mg/kg	14 weeks	Inhibition of cystic growth, and increase of survival	Inhibition of cAMP pathway

*ADPKD* autosomal dominant polycystic kidney disease, *CFTR* cystic fibrosis transmembrane conductance receptor, *PCK* polycystic kidney disease, *WPK* wistar polycystic kidney, *EGFR* epidermal growth factor receptor

^^Doses are in per kilogram of body weight and frequency of dose is per day

^This is not the exact mechanism, but the suggested mechanism as mentioned in the study

^¥^Cystic burden can also be termed as cystic size

### Clinical evidence

There is only one clinical study (NCT02697617) which is an ongoing phase II study that is targeted to evaluate the effect of the low dose of pioglitazone (15 mg) on the treatment of ADPKD. This study is placebo-controlled, and randomized, and was expected to be completed in October 2020. [ClinicalTrial] We did not find any other clinical study (ongoing/completed) in the literature that is evaluating the efficacy and safety parameters of pioglitazone in ADPKD patients.

## Safety perspectives

The ADPKD is a form of chronic kidney disease (CKD) that leads to reduced kidney function, kidney failure, cardiovascular outcomes, and often warrant dialysis or kidney replacement (Bergmann et al. 2018). In the medical literature related to CKD, although not primarily focused on ADPKD, pioglitazone has been found to be safe, and adjustment of dose is not needed in patients with CKD because there is no accumulation of metabolites of pioglitazone; M3 and M4 (Satirapoj et al. 2018; Budde et al. 2003). Although rare but the only serious adverse drug reaction (ADR) related to pioglitazone is urinary bladder cancer which the patients and physicians need to be aware. Furthermore, this ADR is mainly dose and time-dependent (Zmily et al. 2011). Satirapoj et al. investigated the effect of low dose (7.5 mg/d) and standard dose (15 mg/d) of pioglitazone in patients with T2DM and chronic kidney disease. They found that the standard dose led to a greater increase in fluid retention, extracellular water composition, and body weight but no serious adverse drug reactions like hypoglycemia, abnormal liver function, and congestive heart failure were identified in both the groups (Satirapoj et al. 2018). Another study reported the effect of pioglitazone on the cardiovascular outcomes in patients with diabetes and CKD. Schneider et al. in this study reported that patients were found to be less likely to reach the primary composite endpoint (myocardial infarction, acute coronary syndrome, stroke, and all-cause mortality) that again confers that pioglitazone is indeed a safe drug (Schneider et al. 2008). Furthermore, as per the product monograph; summary of product characteristics (SmPC) of pioglitazone; dose adjustment is not needed in the patients with renal impairment (creatinine clearance greater than 4 ml/min) which as per the Grantham et al. in ADPKD patients is around 70 ml/min (Compendium and Actos Tablets 2020; Grantham and Torres 2016).

## Rationality behind the use of pioglitazone as a therapeutic option for ADPKD

**Fig. 5:**
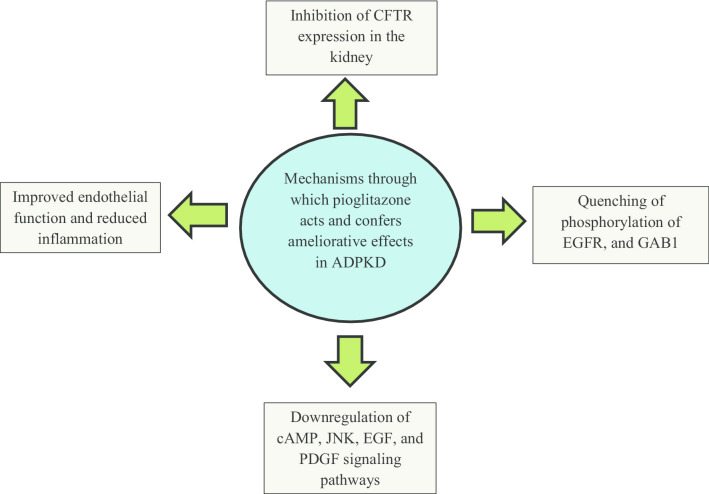
Different mechanisms through which pioglitazone confers its actions in ADPKD

To date, in different preclinical studies, this PPAR-γ agonist, pioglitazone, has been shown to provide anti-cystogenic properties through multiple mechanisms that inarguably suggests its pleiotropy (Fig. 5). The above-mentioned successive preclinical studies published so far confer the data that are compelling enough to validate the fact that pioglitazone treatment has the potential to help attenuate the cystic growth and the effects of this devastating disorder. In the study of Raphael et al., we see that pioglitazone increased the survival of mice which was 90.2 ± 11.5 days as compared to the control group in which it was 57.2 ± 4.9 days with a statistically significant difference of P = 0.034 (Raphael et al. 2009b).

CFTR, a chloride ion channel, encoded by the CFTR gene whose mutation has been linked to be responsible for the fluid secretion by the thin-walled epithelium of cyst in ADPKD (Sun et al. 2011). In a breakthrough study of Blazer-Yost et al., we can easily make an inference that CFTR ion channel expression was reduced when preclinical models were administered with pioglitazone, and this too was statistically significant (P = 0.009). A remarkable point that must be considered is that in this study, the renal cystic burden was found to be reduced both at a low dose (7 mg/kg), and a high dose (20 mg/kg). Now, why it is important to emphasize the above-mentioned is because in a chronic disorder like ADPKD, long-term pharmacological therapy is required, so, whatever be the treatment drug, it should have minimal ADR, and with a drug like pioglitazone, this can be ensured as is seen in the above-mentioned study (Blazer-Yost et al. 2010). In a study by Yanai et al., a low dose of pioglitazone that is 7.5 mg/day, was found to show the same improvements in insulin sensitivity as the standard dose (15 mg/day), and high dose (30 mg/day). Moreover, adverse effects related to low-dose pioglitazone like weight gain, and edema was also less as compared to its standard and high dose (Yanai and Adachi 2017). It is well-known that reduced doses of drugs can help prevent ADR and, if pioglitazone can confer the efficacy at low doses, then, it adds advantage and uplifts the ADPKD treatment by pioglitazone.

Another most interesting, and the noteworthy point is that pioglitazone interferes with the expression of CFTR in the cell membrane, but it does not impede the vasopressin action on the water transport or cause polyuria which is very well associated with V2R antagonist such as tolvaptan (Zmily et al. 2011).

In the pathophysiology section of this review, it can be seen that there is a significant scientific consensus that the alteration of different molecular signaling pathways takes place in ADPKD. GSEA showed that pioglitazone can down-regulate the signaling pathways that are concerned with the regulation of cell proliferation, namely, JNK, PDGF, and EGF. Likewise, the gene named SCD1, which plays an important role in cell proliferation, can also get down-regulated by pioglitazone as evidenced by KEGG analysis (Yoshihara et al. 2012). Thus, again proving its versatility in ADPKD treatment.

As mentioned earlier, tolvaptan is the only drug that is approved by the USFDA for the treatment of ADPKD patients (Bergmann et al. 2018). However, its association with adverse effects like chest pain, polyuria, occasional liver injury, and ventricular tachycardia often leads to its discontinuation (Zmily et al. 2011). Although not in the ADPKD patients but pioglitazone, in this regard, is way better than tolvaptan because the associated ADRs like edema and liver injury are dose and time-related in patients with T2DM.

Nevertheless, the efficacy and safety of pioglitazone have not been yet verified in the patients with ADPKD as the only clinical trial (ClinicalTrials.gov number, NCT02697617) assessing the efficacy and safety parameters of pioglitazone in ADPKD patients is still ongoing Clinicaltrials 2020 and in the literature, there are only the preclinical studies (Blazer-Yost et al. 2010; Yoshihara et al. 2012; Flaig et al. 2016; Raphael et al. 2009b; Kanhai et al. 2020; Muto et al. 2002) based on which we can validate its exceptional, and beneficial effects in ADPKD that certainly makes it an outstanding contender among the drugs that are currently being tested under the clinical trials for the treatment of ADPKD. At last, all eyes are on the regulatory body (USFDA) which will base its decision on the data that will come after the completion of the above-mentioned trial after which the rationality behind the pioglitazone treatment in ADPKD patients will get more strengthened and apparent.

## Conclusion

Pioglitazone, a PPAR-γ agonist, is not only a major therapeutic agent to treat T2DM but is an upcoming drug to treat the potentially lethal ADPKD. Its efficacy in preclinical studies has made it an efficient drug for ADPKD treatment. Though there are several molecular mechanisms of cystogenesis in ADPKD that have been ascertained, but the exact mechanism is still not completely understood which definitely warrants further studies. This is also true for the mechanisms of pioglitazone through which it exhibits such commendable treatment effects in ADPKD as it is justifiable from the review that to date, there are only six preclinical studies related to pioglitazone for ADPKD treatment. Last, it can be stated hopefully that pioglitazone will get approval from the regulatory body once the ongoing clinical trial (NCT02697617) is completed.

## Data Availability

Not applicable.

## Abbreviations

ADPKD: Autosomal dominant polycystic kidney disease
PKD: Polycystic kidney disease
FDA: Food and drugs administration
USA: United States of America
V2R: Vasopressin receptor-2
LVH: Left ventricular hypertrophy
MRI: Magnetic resonance imagining
LVMI: Left ventricular mass index
SMO: Smoothened antagonist
PCP: Planar cell polarity
cAMP: Cyclic adenosine monophosphate
CT: Computed tomography
ciPTEC: Conditionally immortalized proximal tubular epithelial cells
DNA: Deoxy-ribonucleic acid
JAK: Janus kinase
STAT: Signal transducer and activator of transcription
MEK: Mitogen-activated protein kinase
RAF: Rapidly accelerated fibrosarcoma
ERK: Extracellular signal-regulated kinase
MAPK: Mitogen-activated protein kinase
CFTR: Cystic fibrosis transmembrane conductance receptor
ESRD: End stage renal disease
EGF: Epidermal growth factor
EFGR: Epidermal growth factor
SMPC: Summary of product characteristics
AKT: Protein kinase B
PI3K: Phosphatidylinositol-3-phosphate
ER: Endoplasmic reticulum
AAA: Abdominal aortic aneurysm
mTOR: Mammalian target of rapamycin
WPK: Wistar polycystic kidney
PPAR: Peroxisome-proliferator activated-receptor gamma
KEGG: Kyoto encyclopedia of gene and genomes
GSEA: Gene set enrichment analysis
PECAM-1: Platelet endothelial cell adhesion molecule-1
